# Associations between the social environment and early childhood developmental outcomes of Puerto Rican children with prenatal Zika virus exposure: a cross-sectional study

**DOI:** 10.1186/s12887-024-04806-y

**Published:** 2024-05-17

**Authors:** Mary Rodríguez-Rabassa, Allison A. Appleton, Viviana Rosario-Villafañe, Irelis Repollet-Carrer, Marilyn Borges-Rodríguez, Lydiet Dedós-Peña, Marielly González, Paola Velázquez-González, Kamalich Muniz-Rodriguez, Claudia Mántaras-Ortiz, Vanessa Rivera-Amill, Odette Olivieri-Ramos, Luisa I. Alvarado-Domenech

**Affiliations:** 1 Department of Pediatrics, Ponce Health Sciences University, Ponce, PR Puerto Rico; 2 RCMI Center for Research Resources, Ponce Health Sciences University, Ponce, PR Puerto Rico; 3 Department of Epidemiology and Biostatistics, University at Albany School of Public Health, State University of New York, Rensselaer, NY USA; 4 Ponce Research Institute, Ponce Health Sciences University, Ponce, PR Puerto Rico; 5 Clinical Psychology Program, Ponce Health Sciences University, Ponce, PR 00732-7004 Puerto Rico

**Keywords:** Socioenvironmental characteristics, Congenital Zika virus exposure, Prenatal Zika virus exposure, Children without microcephaly/asymptomatic at birth, Neurodevelopmental outcomes

## Abstract

**Background:**

Prenatal exposure to the Zika virus can lead to microcephaly and adverse developmental outcomes, even in children without evident birth defects. The social environment plays a crucial role in infant health and developmental trajectories, especially during periods of heightened brain plasticity. The study aimed to assess socioenvironmental factors as predictors of developmental outcomes of 36-month-old children exposed to Zika virus prenatally.

**Study design:**

This cross-sectional study included 53 mothers and 55 children enrolled in the Pediatric Outcomes of Prenatal Zika Exposure cohort study in Puerto Rico. The study performs follow-up developmental assessments of children born to mothers with confirmed and probable Zika virus infection during pregnancy. Mothers completed socioenvironmental questionnaires (e.g., Perceived Neighborhood Scale and US Household Food Insecurity Survey). Children’s developmental outcomes were assessed with the Bayley Scales of Infant and Toddler Development: Third Edition, the Ages and Stages Questionnaires: Third Edition, the Ages and Stages Questionnaire-Socioemotional: Second Edition, and the Child Adjustment and Parent Efficacy Scale.

**Results:**

Linear regression models, adjusting for a child’s sex and age and maternal education, revealed that early life exposure to food insecurity and maternal pregnancy stressors were significantly associated with poorer developmental outcomes in Zika virus-exposed children at 36 months of age. Maternal resilience representation of adaptive ability was associated with the preservation of adequate developmental outcomes in children.

**Conclusions:**

Pregnancy and early childhood are critical life periods for ensuring optimal brain development in children. While the mechanisms in the interaction of children with their environment are complex, the risk and protective factors identified in the study are modifiable through public policy and preventive initiatives. Implementation of comprehensive strategies that improve access to social support programs, educational and nutritional interventions, and mental health services during pregnancy and early childhood can enhance the developmental potential of vulnerable children.

**Supplementary Information:**

The online version contains supplementary material available at 10.1186/s12887-024-04806-y.

## Introduction

The environment that surrounds and interacts with children offers opportunities for growth, development, and play that have long-lasting effects on their overall health and well-being. Experts attribute a key role to a stable and responsible caregiving environment in driving normative brain and behavioral development [1]. In fact, relational health in stable, nurturing, and safe environments has been confirmed to mitigate the effects of chronic hardship, which can ensue from disadvantaged social determinants of health, poverty, maternal stress, and food insecurity [2]. In addition, a child’s neighborhood environment is recognized for its critical direct and indirect influences on child health during sensitive life periods from fetal life to early childhood [3]. Protective factors and risks of the early environment interact and contribute to child health, development, and future health disparities through complex mechanisms, including epigenetic, neurohormonal, and relational mechanisms, that merit further study to inform healthcare and public health policy [4].

Congenital infections disrupt neuronal processes through direct infection of developing brain cells, exposure to inflammatory cytokines during maternal infection, and direct damage to the placenta, leading to poor perfusion and malfunction that alters fetal development [5]. Zika virus infection during pregnancy has been associated with congenital Zika syndrome microcephaly and severe brain defects in infants, as well as neurodevelopmental concerns that develop over time in those without detectable birth defects [6–9]. However, brain plasticity during early childhood provides a window of opportunity for stimulating brain development through interactions with the environment and adaptations based on learning and experiences. These dynamic interactions are influenced by socioenvironmental factors, which have not been thoroughly analyzed as risk or protective factors for developmental outcomes in children with prenatal Zika exposure.

Since the 2016-17 Zika epidemic in Puerto Rico (PR), the Pediatric Outcomes of Prenatal Zika Exposure (POPZE) study systematically followed a cohort of Hispanic children with exposure. Close developmental monitoring and assessments have revealed that at least one-third of the children at 36 months of age have developmental delays in at least one domain (cognitive, language, or motor), with a reduced prevalence of delays noted in some domains over time [6]. Data on developmental delays in Puerto Rican children 0 to 5 years are limited. However, the prevalence of delay in the study children is notably higher than the 2018–2019 US prevalence for emotional, developmental, and behavioral problems of 10.8% among children aged 0–5 years [10]. The occurrence of delay in the language domain decreased from 50.9% at 24 months to 31.9% at 36 months of age. This prevalence is similar to the combined prevalence of 29.7% reported in a recent systematic review by Marbán-Castro et al. [11]. among normocephalic children with prenatal Zika virus exposure. This manuscript provides updated results of developmental outcomes from 55 children at 36 months of age with prenatal Zika virus exposure and explores socioenvironmental factors as predictors of such outcomes.

## Methods

Participants in this cross-sectional study include children and mothers enrolled in the POPZE cohort study in Puerto Rico between May 2017 and December 2019 with follow-up developmental assessments at 36 months. The study included professional assessments and parental perspectives on the children’s development and collected socioenvironmental risk and protective factors with an impact on childhood development at the 36-month visit (from September 2019 to March 2021) (see Supplementary Table 1 for detailed information on the timing of assessment and response rate for each instrument and Supplementary Table 2 for psychometric properties of the tools).

The original phase of the study, POPZE I, aimed to characterize the full spectrum of structural and functional abnormalities in children born to mothers infected with ZIKV infection during pregnancy. From May 2017 to June 2019, the team enrolled 114 exposed children and followed 97 of them. With a new funding source starting in August 2019, the team expanded its focus in POPZE II to assess the interaction of socioenvironmental aspects with the exposed children’s development. This phase enrolled the mothers as active participants, with 58 out of 97 (59.8%) re-enrolled for the expanded follow-up visits. The re-consent period took place during the follow-up visits beginning in August 2019. Fifty-five out of 58 (94.8%) participants enrolled in POPZE II completed the assessment of the 36-month visit. Three children were excluded because they did not attend the 36-month scheduled appointment. Participants’ enrollment, inclusion, and exclusion criteria were previously described in a manuscript by Alvarado-Domenech et al. [6]. Briefly, children were born at two academic hospitals in the southern region of Puerto Rico, and their mothers had confirmed (polymerase chain reaction (PCR) positive) or probable (immunoglobulin M (IgM) positive) Zika virus infection. The Ponce Medical School Foundation Institutional Review Board reviewed and approved this study. All parents provided informed written consent for both their own and their child’s participation in the study.

### Developmental outcomes

Experienced licensed clinical psychologists administered the Bayley Scales of Infant and Toddler Development, Third Edition (BSID-III) [12] in Spanish to assess the children’s neurodevelopmental functioning based on cognitive, language, and motor domains at 36 months of age. Percentile scores describe the child’s performance in each domain; higher scores imply higher functioning. Parents answered the Ages and Stages Questionnaire, Third Edition (ASQ-3) [13], providing perceptions on their child’s development in communication, gross motor, fine motor, problem solving, and personal-social skills. Higher scores imply better execution on the assessed domain. Parents also completed the Ages and Stages Questionnaire: Social-Emotional, Second Edition (ASQ: SE-2) [14], which assesses children’s responses indicating risk for social or emotional difficulties. Higher scores imply higher social or emotional difficulties. These developmental tests have been widely used to detect early developmental delay or risk of delay in other Zika virus cohorts [6, 7, 15–17], allowing comparison with other populations.

The Child Adjustment and Parent Efficacy Scale (CAPES) [18, 19] assesses children’s behavioral and emotional difficulties through the Behavioral and Emotional Adjustment scales, respectively. These scales are combined in the intensity scale, which describes the parent’s perception on the children’s psychological difficulties. The CAPES also assesses parent’s perception of their self-efficacy in managing their child’s behavioral and emotional problems through the Parent Self-Efficacy scale. Some items are reverse scored. Higher scores indicate a higher level of the construct measured.

### Socioenvironmental factors

Neighborhood characteristics include the Area Deprivation Index (ADI) [20, 21] and the Perceived Neighborhood Scale (PNS) [22]. The team used the Neighborhood Atlas platform to obtain the participants’ home ADI, an index that ranks the neighborhood based on US Census-identified socioeconomic disadvantage [20, 21]. Participants’ physical addresses were entered on Puerto Rico’s map to obtain the national percentile rank for each participant. A higher percentile score denotes a more disadvantaged neighborhood. Also, the team used a cutoff percentile of 80 to classify families living in higher disadvantage areas. The PNS assesses the parental perception of a neighborhood within four dimensions: social embeddedness, sense of community, neighborhood satisfaction, and perceived crime [22]. Higher scores imply a higher degree of the dimension measured.

Home and family characteristics include the Home Observation Measurement of the Environment – Short Form (HOME-SF) [23], the McMaster Family Assessment Device-Short Form (FAD-SF) [24, 25] and the United States Department of Agriculture (USDA) – Household Food Security Survey Module (HFSSM) [26], all completed by the mothers. The HOME-SF assesses the quality of the cognitive stimulation (e.g., “How often do you read stories to your child?”) and emotional support (e.g., “Does your child see his/her father or father-figure on a daily basis?”) in the child’s home environment [23]. Higher scores indicate a better home environment. The FAD-SF assesses family functioning (e.g., “We can express feelings to each other”) [24]. Higher scores indicate worse functioning [27]. The USDA – HFSSM assesses household food security. Parents reported their access to food of sufficient quantity and quality for the last 12 months. Based on the USDA – HFSSM interpretation scale [26], a score of 1 is considered marginal food insecurity, suggesting a family is vulnerable to food insecurity. Higher scores indicate higher food insecurity.

Maternal characteristics include the following self-report instruments: Zika Virus-Related Prenatal Stress Scale (ZIKV-PSS; developed by researchers; α = 0.89), Beck Depression Inventory (BDI-II) [28], Maternal Resilience Scale (ERESMA) [29], and Social Provisions Scale (SPS) [30]. The ZIKV-PSS assesses stress during pregnancy, including Zika virus-related stressors (e.g., “Receive a Zika diagnosis,” “Not having family support”). To reduce recall bias, mothers were asked to make an effort to place themselves at the stage of pregnancy. The BDI-II explores depression symptomatology (e.g., “Sadness”). ERESMA assesses the resilience of mothers of exceptional children (e.g., “I try to make sure that my child is happy”) [29]. The SPS evaluates perceived social support (e.g., “There are people I can count on in an emergency”) [30]. For each instrument, higher scores indicate a greater degree of the construct assessed.

### Statistical analyses

Descriptive statistics were calculated for sociodemographic and socioenvironmental factors and developmental outcomes of the participants in the study. For continuous variables, the mean (SD) was calculated, while percentages were used for categorical variables. Socioenvironmental factors and developmental outcome scores were analyzed as continuous variables. Maternal education was categorized into two groups: (1) high school or lower and (2) higher than high school, representing the highest level of education achieved. Linear regression models were employed, adjusting for child sex, age, and maternal education to examine the associations between each socioenvironmental characteristic and each developmental outcome; *P* values of < 0.05 denoted statistical significance. Analyses were performed with SAS version 9.4 (SAS Institute).

## Results

The sociodemographic and clinical characteristics of the mother and child are presented in Table 1. Fifty-three mothers of 55 children, including two sets of twins, completed the study questionnaires accounting for a 100% response rate. The maternal mean age was 30 years. Of the mothers, 49.1% (26) were unemployed, and 77.4% (41) had an educational level of more than high school, of which 31.7% (13) had technical certificates and 34.2% (14) had associate degrees. Most women were married or cohabitating (*n* = 40; 75.7%), had public health insurance (*n* = 41; 77.4%), and had an annual household income <$15,000 (*n* = 38; 71.7%). Zika virus infection during pregnancy was documented in the first (*n* = 22; 41.5%), second (*n* = 18; 33.9%), and third (*n* = 13; 24.5%) trimesters. Maternal diagnostic tests indicated: PCR positive (*n* = 16; 30.2%), IgM positive (*n* = 27; 50.9%), or both (*n* = 10; 18.9%) assays results. Of the children enrolled in this study phase, none (0%) had microcephaly (head circumference *z*-score < -2), 2 of 53 (3.8%) had abnormal hearing screening, and 11 of 17 (64.7%) had retinal images abnormalities at birth. The children’s mean age at the 36-month assessment was 36.6 months, and 56.4% (31) were female.

**Table 1:** Maternal and child characteristics and socioenvironmental profile

Characteristics	*n*/*N* (%)	Mean (SD)	Range	
Sociodemographic/clinical				
Maternal				
Age, years		30.07 (6.19)	16–43	
Married/cohabitating	40/53 (75.74)			
Health insurance, public	41/53 (77.36)			
Educational attainment				
LTHS or HS	12/53 (22.64)			
More than HS	41/53 (77.36)			
Technical certificate	13/41 (31.71)			
Associate degree	14/41 (34.15)			
Bachelor’s degree	11/41 (26.83)			
Master’s degree	3/41 (7.32)			
Unemployment	26/53 (49.06)			
Household income < $15,000	38/53 (71.70)			
Residential area, rural	19/53 (35.85)			
Prenatal Zika virus diagnosis				
PCR positive	16/53 (30.19)			
IgM positive	27/53 (50.94)			
Both positive	10/53 (18.87)			
Trimester of symptomatic infection				
First	22/53 (41.51)			
Second	18/53 (33.96)			
Third	13/53 (24.53)			
Child				
Age, months		36.58 (1.04)	35.03–39.83	
Female, sex	31/55 (56.36)			
Microcephaly^†^, HC *z-*score *≤*-2	0/55 (0.0)			
Abnormal ABR^†^	2/53 (3.77)			
Abnormal RetCam image^†^	11/17 (64.71)			
Socioenvironmental profile				
Neighborhood				
ADI, more disadvantage, *≥* 80th percentile	48/53 (90.57)	89.38 (11.14)	51–100	
Perceived Neighborhood Scale				
Social embeddedness		25.00 (9.34)	9–43	
Sense of community		26.40 (6.99)	7–35	
Satisfaction		36.87 (6.55)	18–45	
Perceived crime		16.11 (8.12)	9–45	
Home and family				
HOME cognitive stimulation		2.93 (1.02)	1–6	
HOME emotional support		2.89 (0.98)	1–5	
McMaster family functioning		17.33 (6.50)	12–45	
Food insecurity vulnerability	21/53 (39.62)	1.40 (2.85)	0–15	
Maternal characteristics				
Prenatal stress		31.11 (12.22)	6–59	
Depression		8.84 (9.25)	0–33	
Resilience		211.16 (13.12)	169–225	
Social Support		89.93 (11.76)	33–96	

*HS*, high school; *LTHS*, less than high school; *ADI*, area deprivation index (national); *HOME*, home observation measurement of the environment; *PCR, polymerase chain reaction; IgM, immunoglobulin M; HC*, head circumference; *ABR*, Automated Auditory Brainstem Response; *RetCam*, Retinal imaging with RetCam 3 Visualization System.

^†^At birth

### Socioenvironmental factors

The ADI neighborhood measure revealed a mean percentile rank of 89.4 (SD, 11.1), indicating that most participants (90.6%) live in highly disadvantaged areas (Table 1). However, maternal reports on the PNS revealed mean scores that suggest they perceive an average social embeddedness (25.0; SD, 9.3), a high sense of community (26.4; SD, 6.6) and satisfaction (36.9; SD, 6.6), and a low perceived crime (16.1; SD, 8.1). Regarding the home and family characteristics, parental mean scores on the HOME cognitive stimulation (2.9; SD, 1.0) and emotional support (2.9; SD, 0.98) scales were average (Table 1). Additionally, the mean score (17.3; SD, 6.5) on the FAD-SF scale revealed adequate family functioning. The household food security mean score (1.4; SD, 2.9) suggested that about one-third (21/53; 39.6%) of these families experienced food insecurity vulnerability, a rate disproportionally higher than the prevalence of 13.3% and 6% among the households of US children with and without special healthcare needs, respectively [31]. Regarding maternal characteristics, maternal perceived stress during pregnancy varied (range, 6–59), with a moderate mean score (31.1; SD, 12.2) on the prenatal stress scale (Table 1). Depression symptomatology on the BDI-II mean score was low (8.8; SD, 9.3). Mean scores on the ERESMA scale (211.2; SD, 13.1) suggested that these women manifest high levels of resilience. Additionally, mean scores on the SPS (89.9; SD, 11.8) reflected that they perceive excellent sources of social support (e.g., having a person to rely on when facing difficulties and counting on individuals who recognize and appreciate their skills and abilities).

### Developmental outcomes

At 36 months of age, the mean percentile of children’s neurodevelopment scores in the BSID-III indicate average performance in all domains: cognitive (34.5; SD, 17.3), language (29.2; SD, 23.4), and motor (30.8; SD, 21.4) (Table 2). Mean scores on the ASQ-3 were average for communication (44.8; SD, 18.2), gross motor (55.4; SD, 6.6), and fine motor (40.3; SD, 18.1) skills domains. However, parental reports showed below average scores on problem solving (37.2; SD, 15.1) and personal-social (43.2; SD, 14.9) domains, both in the “monitoring zone”, indicating that the children could benefit from further follow-up developmental assessments.

**Table 2:** Child neurodevelopmental assessment scores and prevalence of developmental delay/risk according to BSID-III, ASQ-3, ASQ: SE-2, and CAPES

Outcomes	Mean (SD)	Range	^†^Delay or Risk *n*/*N* (%)	^‡^*P*-value
BSID-III, percentiles				
Cognitive	34.52 (17.29)	5–91	5/52 (9.62)	0.58
Language	29.22 (23.40)	0–92	18/52 (34.62)	0.30
Motor	30.83 (21.37)	0–92	15/52 (28.85)	0.84
ASQ-3				
Communication	44.80 (18.17)	0–60	19/51 (37.25)	0.13
Gross motor	56.37 (6.64)	30–60	4/51 (7.84)	0.22
Fine motor	40.29 (18.08)	0–60	14/52 (26.92)	0.63
Problem solving	37.16 (15.11)*	0–60	24/51 (47.06)	0.25
Personal-social	43.24 (14.89)*	5–60	23/51 (45.10)	0.95
ASQ: SE-2	54.26 (32.63)	10–165	12/54 (22.22)	0.74
CAPES (*n* = 55)				
Intensity	18.93 (11.03)	3–51	n/a	0.09ˆ
Emotional	1.64 (2.09)	0–8	n/a	0.56ˆ
Behavior	17.29 (9.39)	3–44	n/a	0.07ˆ
Self-efficacy	168.95 (27.33)	70–190	n/a	0.19ˆ

*Scores within the monitoring zone

^**†**^BSID-III, based on *≥* 1 standard deviation below average (composite score cutoff of < 85 or the equivalent of < 16th percentile); ASQ-3 and ASQ: SE-2, based on age-specific cutoff score; CAPES scores are analyzed as a continuous variable, higher scores indicate a higher level of the construct measured

^‡^Pearson chi-square test comparing frequency of delays according to trimester of infection

ˆKruskal Wallis test comparing scores according to trimester of infection

Parental reports on the ASQ: SE-2 displayed average scores (54.3; SD, 32.6), suggesting adequate socioemotional development. This was supported by low mean scores on the CAPES intensity (18.9; SD, 11.0), emotional (1.6; SD, 2.1), and behavior (17.3; SD, 9.4) scales. Additionally, mean scores on the CAPES self-efficacy scale (168.9; SD, 27.3) suggested a high level of perceived parental ability to manage their children’s emotional and behavioral problems. On further analyses, no significant differences were found based on trimester of exposure.

### Associations between children’s developmental outcomes and socioenvironmental factors

Linear regression models, adjusted by age, child’s sex, and maternal education, were used to explore associations between BSID-III, ASQ-3, ASQ: SE-2, and CAPES outcomes and socioenvironmental factors (Tables 3, 4 and 5). Multiple socioenvironmental factors showed significant associations with child developmental outcomes, but the most salient findings indicated that food insecurity and maternal prenatal stress are linked to poorer developmental outcomes. However, maternal resilience stood out positively, revealing a link with better child outcomes. The following paragraphs illustrate the results of the linear regression analyses by each child’s outcome.

**Table 3:** Associations between socio-environmental characteristics and BSID-III scores at 36 months (B, SE)*

	BSID-III percentile score^±^								
	**Cognitive**			**Language**			**Motor**		
	**B (SE)**	***p***		**B (SE)**	***p***		**B (SE)**	***p***	
Neighborhood charactersistics^δ^									
Area deprivation index, national	-0.13 (0.20)	0.53		-0.51 (0.26)	0.05		-0.03 (0.25)	0.92	
Perceived Neighborhood Scale									
Social embeddedness	0.01 (0.26)	0.96		-0.28 (0.34)	0.41		-0.12 (0.31)	0.71	
Sense of community	0.36 (0.36)	0.32		-0.29 (0.47)	0.54		0.12 (0.44)	0.78	
Satisfaction	0.41 (0.38)	0.28		-0.09 (0.50)	0.86		0.41 (0.47)	0.38	
Perceived crime	-0.30 (0.29)	0.31		-0.43 (0.38)	0.27		-0.65 (0.35)	0.07	
Home and family characteristics^δ^									
HOME cognitive stimulation	1.29 (2.40)	0.59		4.51 (3.07)	0.15		3.36 (2.89)	0.25	
HOME emotional support	1.50 (2.50)	0.55		-3.81 (3.24)	0.25		-2.54 (3.05)	0.41	
McMaster family functioning	-0.35 (0.36)	0.34		-0.12 (0.48)	0.80		0.14 (0.45)	0.75	
Food insecurity	-1.62 (0.77)	0.04		-1.13 (1.04)	0.28		-1.57 (0.96)	0.11	
Maternal characteristics									
Prenatal stress^†^	0.32 (0.20)	0.11		-0.79 (0.23)	0.01		-0.59 (0.24)	0.02	
Depression^δ^	-0.33 (0.26)	0.21		-0.10 (0.34)	0.78		0.01 (0.32)	0.96	
Resilience^δ^	0.48 (0.19)	0.01		0.52 (0.04)	0.24		0.34 (0.24)	0.15	
Social support^δ^	0.10 (0.21)	0.62		0.03 (0.27)	0.92		-0.20 (0.24)	0.43	

*All linear regression models are adjusted for age, child sex, and maternal education.

^±^*n* = 52; ^δ^*n* = 55; ^†^*n* = 54.

**Table 4:** Associations between socio-environmental characteristics and ASQ-3 / ASQ: SE-2 scores at 36 months (B, SE)*

	ASQ-3 Score																					ASQ: SE-2 Score			
	Communication			Gross Motor			Fine Motor					Problem Solving			Personal-Social						Social-Emotional				
	B (SE)	*p*		B (SE)	*p*		B (SE)	*p*			B (SE)		*p*		B (SE)	*p*				B (SE)			*p*		
Neighborhood charactersistics																									
Area deprivation index, national	-0.02 (0.24)	0.93		-0.07 (0.10)	0.50		-0.14 (0.24)	0.55			-0.09 (0.20)		0.65		0.06 (0.17)	0.61				0.26 (0.35)			0.47		
Perceived Neighborhood Scale																									
Social embeddedness	0.25 (0.29)	0.40		0.14 (0.12)	0.26		0.31 (0.28)	0.28			0.09 (0.21)		0.73		0.51 (0.21)	0.81				-0.43 (0.44)			0.33		
Sense of community	0.29 (0.43)	0.50		-0.03 (0.18)	0.88		0.21 (0.42)	0.62			-0.07 (0.36)		0.84		0.05 (0.31)	0.87				-0.79 (0.60)			0.19		
Satisfaction	0.14 (0.44)	0.74		0.06 (0.18)	0.73		0.09 (0.43)	0.83			-0.12 (0.37)		0.75		0.37 (0.31)	0.23				-0.91 (0.69)			0.20		
Perceived crime	-0.64 (0.37)	0.09		-0.07 (0.16)	0.67		-0.17 (0.37)	0.66			-0.11 (0.32)		0.73		-0.64 (0.25)	0.02				0.32 (0.52)			0.54		
Home and family characteristics																									
HOME cognitive stimulation	4.15 (2.53)	0.10		-1.49 (1.03)	0.16		1.95 (2.53)	0.45			1.51 (2.19)		0.49		1.83 (1.85)	0.32				0.52 (4.0)			0.89		
HOME emotional support	-1.04 (2.78)	0.71		-0.79 (1.13)	0.48		-3.11 (2.72)	0.26			-3.30 (2.29)		0.16		-0.12 (1.98)	0.95				-2.24 (4.24)			0.60		
McMaster family functioning	-0.09 (0.34)	0.81		0.03 (0.16)	0.88		0.16 (0.39)	0.67			0.02 (0.34)		0.95		-0.11 (0.28)	0.70				1.12 (0.66)			0.09		
Food insecurity	-1.73 (0.80)	0.04		-0.49 (0.34)	0.16		-1.53 (0.80)	0.06			-1.11 (0.69)		0.12		-1.63 (0.54)	0.005				4.28 (1.73)			0.02		
Maternal characteristics																									
Prenatal stress	-0.32 (0.22)	0.16		-0.05 (0.09)	0.60		-0.19 (0.21)	0.40			-0.30 (0.18)		0.11		-0.36 (0.15)	0.02				0.73 (0.05)			0.04		
Depression	-0.14 (0.29)	0.64		-0.01 (0.12)	0.92		-0.18 (0.28)	0.53			-0.15 (0.24)		0.55		-0.25 (0.21)	0.23				0.48 (0.37)			0.21		
Resilience	0.36 (0.20)	0.08		-0.02 (0.09)	0.82		0.36 (0.20)	0.08			0.34 (0.17)		0.05		0.31 (0.14)	0.04				-1.02 (0.32)			0.002		
Social support	-0.07 (0.22)	0.74		-0.05 (0.09)	0.57		-0.17 (0.21)	0.42			0.01 (0.19)		0.96		-0.06 (0.15)	0.69				-0.45 (0.35)			0.20		

*All linear regression models are adjusted for age, child sex, and maternal education

Children who experience higher scores in food insecurity (B = -1.62, SE = 0.77, *p* = 0.04) and reduced maternal resilience (B = 0.48, SE = 0.19, *p* = 0.01) had significantly lower scores in the BSID-III cognitive domain (Table 3). Those with higher maternal prenatal stress scores (B = -0.79, SE = 0.23, *p* = 0.01) and higher ADI percentile ranks, thus living in highly disadvantaged neighborhoods (B = -0.51, SE = 0.26, *p* = 0.05), had significantly lower scores in the BSID-III language domain. Children with higher maternal prenatal stress scores (B = -0.59, SE = 0.24, *p* = 0.02) had significantly lower BSID-III motor domain scores.

Consistently, those children with higher scores in food insecurity (B = -1.73, SE = 0.80, *p* = 0.04) (Table 4) had significantly lower scores in the ASQ-3 communication domain. Those with lower scores in maternal resilience (B = 0.34, SE = 0.17, *p* = 0.05) had significantly lower scores in the ASQ-3 problem-solving domain. Additionally, children with higher scores in neighborhood perceived crime (B = -0.64, SE = 0.25, *p* = 0.02), food insecurity (B = -1.63, SE = 0.54, *p* = 0.005), and maternal prenatal stress (B = -0.36, SE = 0.15, *p* = 0.02) and lower maternal resilience (B = 0.31, SE = 0.14, *p* = 0.04) showed significantly lower scores in the ASQ-3 personal-social domain. Children with higher scores for food insecurity (B = 4.28, SE = 1.73, *p* = 0.02) and maternal prenatal stress (B = 0.73, SE = 0.05, *p* = 0.04) and lower scores for maternal resilience (B = -1.02, SE = 0.32, *p* = 0.02) had significantly higher scores for social-emotional difficulties (ASQ: SE-2).

**Table 5:** Associations between socio-environmental characteristics and CAPES scores at 36 months (B, SE)*

	CAPES score − 36 months					
	Intensity				Self-efficacy	
	B (SE)	*p*			B (SE)	*p*
Neighborhood charactersistics						
Area deprivation index, national	0.07 (0.13)	0.60			-0.06 (0.34)	0.87
Perceived Neighborhood Scale						
Social embeddedness	-0.03 (0.16)	0.85			1.30 (0.38)	0.001
Sense of community	-0.17 (0.21)	0.42			1.62 (0.52)	0.003
Satisfaction	-0.44 (0.22)	0.06			1.27 (0.60)	0.04
Perceived crime	0.15 (0.18)	0.40			-0.40 (0.47)	0.40
Home and family characteristics						
HOME cognitive stimulation	-1.23 (1.42)	0.39			-0.82 (3.82)	0.83
HOME emotional support	-2.99 (1.39)	0.04			0.82 (3.88)	0.83
McMaster family functioning	0.34 (0.22)	0.13			-2.32 (0.58)	< 0.0001
Food insecurity	1.37 (0.46)	0.005			-0.10 (1.33)	0.94
Maternal characteristics						
Prenatal stress	0.36 (0.12)	0.003			-0.70 (0.33)	0.04
Depression	0.48 (0.15)	0.002			-2.08 (0.32)	< 0.0001
Resilience	-0.36 (0.11)	0.002			1.21 (0.28)	< 0.0001
Social support	-0.17 (0.12)	0.17			1.16 (0.30)	0.0003

*All linear regression models are adjusted for age, child sex, and maternal education

The CAPES intensity scale combines the emotional adjustment and behavior scales, thus reflecting emotional and behavioral problems. Children with lower scores on HOME emotional support (B = -2.99, SE = 1.39, *p* = 0.04) and maternal resilience (B = -0.36, SE = 0.11, *p* = 0.002) and higher scores on food insecurity (B = 1.37, SE = 0.46, *p* = 0.005), maternal prenatal stress (B = 0.36, SE = 0.12, *p* = 0.003) and depression symptomatology (B = 0.48, SE = 0.15, *p* = 0.002) had significantly higher scores on the intensity scale (Table 5). Those with higher scores in social embeddedness (B = 1.30, SE = 0.38, *p* = 0.001), sense of community (B = 1.62, SE = 0.52, *p* = 0.003), neighborhood satisfaction (B = 1.27, SE = 0.60, *p* = 0.04), social support (B = 1.16, SE = 0.30, *p* = 0.0003), and maternal resilience (B = 1.21, SE = 0.28, *p* < 0.0001) had significantly higher maternal self-efficacy scores. Conversely, higher scores in family functioning (indicating worst functioning) (B = -2.32, SE = 0.58, *p* < 0.0001) and maternal prenatal stress (B = -0.70, SE = 0.33, *p* = 0.04) and depression symptomatology (B = -2.08, SE = 0.32, *p* < 0.0001) were significantly linked with lower maternal self-efficacy scores.

## Discussion

This study explored socioenvironmental variables as risk and protective factors in the development of 55 Hispanic/Latino children from Puerto Rico whose neurodevelopment is biologically vulnerable from prenatal Zika exposure. The findings presented significant associations between early life exposure to food insecurity and maternal pregnancy stressors in the context of social vulnerabilities with developmental risks in these children, highlighting two environmental impediments to the national goal of eliminating disparities and promoting healthy development and well-being across all life stages for all [32]. However, maternal resilience stood out as a remarkable adaptive ability linked to better children’s developmental outcomes.

Social determinants of health significantly impact overall health, well-being, and quality of life [33]. However, beyond addressing each of the vulnerabilities individually, we must consider the impact of the interrelated experiences of material hardships (food insecurity, housing, medical, etc.) [34] that families face. Nevertheless, participants’ characteristics highlight socioeconomic vulnerabilities strongly associated in other studies with the risk of gross motor [35], fine motor [36], communication [37], cognitive [38, 39], and language [40] delays and emotional problems [38, 41–43]. Participants’ vulnerabilities include high unemployment rates, limited household income, public health insurance, and living in disadvantaged areas. These occur in a critical context of years of economic recession in Puerto Rico. Currently, 55% of Puerto Rican families with children have a household income below the poverty level [44], which is disproportionately higher than the rate in the continental U.S. (14.1%) [45], denoting an increased risk for children not attaining developmental potential due to extreme poverty [46, 47]. In addition, there are inherent characteristics of children with developmental disabilities that put them at risk for disadvantage. For example, children with mental, behavioral, and emotional difficulties more often live in the lowest income categories [48].

Food insecurity may be influenced by income and employment status [49] and can limit a child’s intake of essential nutrients, with implications for poor brain development [50]. Despite the compensation of the Nutrition Assistance for Puerto Rico program, the inflation and elevated cost of living represents challenges to Puerto Rican families and jeopardizes access to adequate food for family consumption. Similar to studies linking child malnutrition and lower cognitive scores [51], children in this study who experienced food insecurity had lower performance in BSID cognitive skills. A 24-month longitudinal study by Milner et al. [52]. determined that food insecurity’s timing, intensity, and duration are associated with lower ASQ communication, social, and gross motor scores. While the food insecurity assessment was from the past 12 months, we also found associations with lower ASQ-3 social and communication scores but not with motor scores. We identified other associations with increased behavioral and emotional problems, consistent with other studies of preschool children [53, 54]. Evidence shows that children with special healthcare needs are twice as likely to be food insecure as children without healthcare special needs [31], possibly due to the higher expenditures on services required by this population. Participants in this study showed a greater vulnerability to food insecurity than children with special healthcare needs, with a prevalence of 41.8% versus 13.3% [31].

The findings highlight food insecurity as a noteworthy risk factor for early childhood development within this vulnerable population, even after adjusting for important confounders. Indeed, Hobbs et al. [51] explored the associations between food insecurity and behavioral and cognitive outcomes in 5-year-old children from disadvantaged families. The analysis involved examining these associations across percentiles of food insecurity distribution (10th, 25th, 50th, 75th, and 90th), while controlling for potential confounders such as maternal depression, parenting stress, and material hardship. They found that both externalizing and internalizing behavioral problems were linked to food insecurity across all percentiles. Despite differences in the methodology used to explore associations, this finding is consistent with the association between food insecurity and behavioral and emotional difficulties in POPZE II children, as measured by the CAPES intensity scale and ASQ: SE-2. Regarding cognitive skills, Hobbs et al. [51] reported variability in the evaluated outcomes and the food insecurity percentiles where associations were identified. Food insecurity was associated with lower receptive vocabulary scores in children beyond the 50th percentile. Conversely, low scores in letter-word identification skills were associated with food insecurity only in children at the 10th percentile. The variability in cognitive outcomes might be due to differences in the measures used to assess cognition. Despite the variability, these findings align with POPZE II findings supporting a negative association between food insecurity and cognitive skills. Difficulties identified could potentially have repercussions for psychosocial problems later in adolescence [55] and adulthood [56] in already developmentally vulnerable children.

Cohorts describing birth and follow-up consequences of prenatal Zika have not extensively explored socioenvironmental factors in association to developmental outcomes to allow for comparisons with the Puerto Rican experience. Nevertheless, poor socioenvironmental conditions are known to increase the health risks of vulnerable populations during epidemics. Inadequate home conditions can facilitate Zika virus transmission, and malnutrition and co-infections in the context of socioeconomic disadvantage can affect immune status and the response to infections [57]. In this sense, the study setting serves to highlight the socioenvironmental experiences of many families and children in different countries impacted by the Zika epidemic.

In this study, mothers experienced prenatal Zika virus-related biopsychosocial stressors in the context of social determinants of health vulnerabilities and disadvantages at the individual, family, and neighborhood levels (e.g., low education attainment and household income, food insecurity, and living in disadvantaged areas), factors that have been strongly associated with developmental and health risks in children [58, 59]. Although prenatal stress data were explored retrospectively, introducing recall bias and possible minimization of symptomatology, the findings support previous literature connecting prenatal stress and suboptimal language [60, 61], motor [62], personal-social [63], social-emotional [64, 65], and behavioral [61, 66] abilities in children. The results can also be linked to accumulated evidence that repeated adverse experiences cause “toxic” stress-induced dysregulated stress responses in the mother that can be transmitted intergenerationally to the offspring with long-term consequences in health, development, and well-being [67, 68].

From a positive perspective, despite biopsychosocial vulnerabilities and their stressors, mothers report the ability to adapt and overcome. Maternal resilience was a protective factor in these children’s cognitive, problem solving, personal-social, social-emotional, and behavioral skills. The buffering effect of maternal resilience on child development in the face of adversity is supported by improved family functioning skills and self-efficacy in handling child behavior. Of note, resilience emerges from the dynamic interplay of multiple processes occurring across and between systems [69]. Despite living in disadvantaged areas, families perceive a high sense of community and neighborhood satisfaction. Individuals tend to be more resilient when they have family, social, and community support [70]. In this context, the interaction of children with these systems is critical in learning and developing resilience skills. The role of families and caregivers and the impact of Zika virus exposure on children and families is considerable and should not be overlooked [71]. A positive environment, modeling from significant others, nurturing caregivers, and responsive caregiving promotes positive adjustment and better developmental outcomes [3, 72, 73].

Study limitations include the cross-sectional design and limited sample size, which reduces the generalization of the results. Additionally, data on maternal prenatal stress were collected retrospectively, introducing a recall bias. The lack of a control group precludes the study team from determining whether identified risks and protective factors are shared among groups or are specific to children exposed to Zika virus prenatally. Furthermore, the attrition from the original cohort, mostly due to natural disasters, might have impacted the capacity to retain children with the more severe outcomes in the cohort. We also acknowledge that the psychometric assessment of the newly developed Zika Virus-Related Prenatal Stress Scale requires further validation analyses.

Despite study limitations, to our knowledge, this study is among the first to contribute to assessing multiple socioenvironmental factors as predictors of developmental outcomes of children exposed to Zika virus prenatally. Through a comprehensive assessment implementing standardized procedures with valid and reliable instruments, the findings also contribute to a greater understanding of the factors that may buffer the adverse impact of the Zika virus on the exposed children’s brain development. Immediate family resilience and community supportive influences can be plausible explanations for improved developmental outcomes in some domains observed over time [6] and may add to the understanding of the absence of statistically significant differences in developmental outcomes between exposed and unexposed children reported by prenatal Zika studies of 18- to 30-month-old children who were asymptomatic at birth [16, 74]. The study will publish results of school readiness assessments of exposed and unexposed children from the same community, thereby addressing whether the exposure status determines significant differences in cognitive functioning and other developmental outcomes in the context of relevant socioenvironmental factors.

## Conclusions

Pregnancy and the first years of a child’s life are vital for optimal brain development, as they are periods of great sensitivity and vulnerability for establishing the structural and functional abilities of the brain. Most of the risk and protective factors identified in this study are modifiable through public policy that supports the implementation of comprehensive strategies such as increasing access to social support programs, enhancing educational and nutritional interventions, and providing mental health support to pregnant women and families. Addressing social determinants of health requires transdisciplinary collaboration from policymakers, community partners, and healthcare providers to ensure maternal and child well-being and better health outcomes. Timely provisions of resources and interventions can reduce inequities and enhance the development potential of children and families.

### Electronic supplementary material

Below is the link to the electronic supplementary material.

Supplementary Material 1

Supplementary Material 2

Supplementary Material 3

## Data Availability

The datasets used and analyzed during the current study are available from the corresponding author upon reasonable request.

## Abbreviations

ABR: Automated Auditory Brainstem
ADI: Area deprivation index
ASQ-3: Ages and Stages Questionnaire, Third Edition
ASQ: SE-2: Ages and Stages Questionnaire: Social-Emotional, Second Edition
BDI-II: Beck Depression Inventory – Second Edition
BSID-III: Bayley Scales of Infant and Toddler Development, Third Edition
CAPES: Child Adjustment and Parent Efficacy Scale
HC: Head circumference
HOME-SF: Home Observation Measurement of the Environment – Short Form
HFSSM: Household Food Security Survey Module
IgM: Immunoglobulin M
PCR: Polymerase chain reaction
RetCam: Retinal imaging with RetCam 3 Visualization System
SPS: Social Provisions Scale
USDA: United States Department of Agriculture
ZIKV-PSS: Zika Virus-Related Prenatal Stress Scale
