# Supplementary material for: Associations between the social environment and early childhood developmental outcomes of Puerto Rican children with prenatal Zika virus exposure: a cross-sectional study
Source: BMC Pediatr. 2024 May 17;24:342. doi: 10.1186/s12887-024-04806-y (PMC11100158; doi:10.1186/s12887-024-04806-y)
Supplement: Supplementary file 1 — Supplementary Material 1 [file 12887_2024_4806_MOESM1_ESM.docx]

**Supplementary Table 1. Assessment tests and questionnaires, timing of assessment, and participant’s response rate for each instrument.**

| **Assessment instrument** | **Timing of assessment** | **Response rate n/N (%)** |
| --- | --- | --- |
| Bayley Scales of Infant and Toddler Development, Third Edition (BSID-III) | 36-month visit | 52/55 (94.5) |
| Ages and Stages Questionnaire – Third Edition (ASQ-3) | 36-month visit | 51/55 (92.7) |
| Ages and Stages Questionnaire: Social-Emotional, Second Edition (ASQ:SE-2) | 36-month visit | 54/55 (98.2) |
| Child Adjustment and Parent Efficacy Scale (CAPES) | 36-month visit | 55/55 (100) |
| Area Deprivation Index (ADI)^†^ | 36-month visit | 53/53 (100) |
| Perceived Neighborhood Scale (PNS)^†^ | 36-month visit | 53/53 (100) |
| Home Observation Measurement of the Environment – Short Form (HOME-SF) | 36-month visit | 55/55 (100) |
| United States Department of Agriculture (USDA) – Household Food Security Survey Module (HFSSM)^†^ | 36-month visit | 53/53 (100) |
| McMaster Family Assessment Device-Short Form (FAD-SF)^†^ | 36-month visit | 53/53 (100) |
| Zika Virus-Related Prenatal Stress Scale (ZIKV-PSS)^†^ | 24, 30, or 36-month visits* | 52/53 (98.1) |
| Maternal Resilience Scale (ERESMA)^†^ | 36-month visit | 53/53 (100) |
| Social Provisions Scale (SPS)^†^ | 36-month visit | 53/53 (100) |

*Note*. Participants were between 36 and 39 months old during the 36-month visit.

^†^The study included 53 mothers, two of whom delivered twins, for a total of 55 children. The same data on maternal and family/household characteristics obtained from the mothers of twins was applied to the analysis of association with the children’s outcomes.

*Assessments were performed between August 2019 and March 2020 during the 24 (n = 12), 30 (n = 27), and 36 (n = 15) month visits.
