# Supplementary material for: Associations between the social environment and early childhood developmental outcomes of Puerto Rican children with prenatal Zika virus exposure: a cross-sectional study
Source: BMC Pediatr. 2024 May 17;24:342. doi: 10.1186/s12887-024-04806-y (PMC11100158; doi:10.1186/s12887-024-04806-y)
Supplement: Supplementary file 2 — Supplementary Material 2 [file 12887_2024_4806_MOESM2_ESM.docx]

**Supplementary Table 2. Psychometric properties of the CAPES, ASQ-3, ASQ:SE-2, PNS, ZIKV-PSS, ERESMA, and SPS tools.**

| **Questionnaire** | **Validity*** | **Reliability** | **Internal consistency** | **Specificity** | **Sensitivity** | **Range** | **Minimum** | **Maximum** |
| --- | --- | --- | --- | --- | --- | --- | --- | --- |
| CAPES [18, 19] |  |  |  |  |  |  |  |  |
| Intensity | - | 0.90 | 0.90 | - | - | 90 | 0 | 90 |
| Emotional | - | 0.74 | 0.96 | - | - | 12 | 0 | 12 |
| Behavior | - | 0.90 | 0.74 | - | - | 78 | 0 | 78 |
| Self-efficacy | - | 0.96 | 0.88 | - | - | 180 | 20 | 200 |
| ASQ-3^ [13] | 93.8% | 0.91 | 0.92 | 92.1% | 82.5% | - | - | - |
| Communication | - | - | 0.66 | - | - | 60 | 0 | 60 |
| Gross motor | - | - | 0.72 | - | - | 60 | 0 | 60 |
| Fine motor | - | - | 0.83 | - | - | 60 | 0 | 60 |
| Problem solving | - | - | 0.78 | - | - | 60 | 0 | 60 |
| Personal-social | - | - | 0.67 | - | - | 60 | 0 | 60 |
| ASQ:SE-2^ [14] | 89.1% | 0.98* | 0.90* | 98.0%* | 82.6%* | 310 | 0 | 310 |
| PNS [22] | - | 0.83-0.85 | - | - | - | 136 | 34 | 170 |
| Social embeddedness | - | 0.80-0.83 | - | - | - | 44 | 11 | 55 |
| Sense of community | - | 0.85-0.86 | - | - | - | 28 | 7 | 35 |
| Neigborhood satisfaction | - | 0.83-0.86 | - | - | - | 28 | 7 | 35 |
| Perceived crime | - | 0.85-0.91 | - | - | - | 36 | 9 | 45 |
| ZIKV-PSS | - | 0.89 | - | - | - | 72 | 0 | 72 |
| ERESMA [29] | - | 0.92 | - | - | - | 180 | 45 | 225 |
| SPS [30] | - | 0.92 | - | - | - | 72 | 24 | 96 |
| *Concurrent validity (agreement between results and professional standarized assessments).  ^Data corresponds to the 60-months questionnaire. | | | | | |  |  |  |
