# Supplementary material for: Associations between the social environment and early childhood developmental outcomes of Puerto Rican children with prenatal Zika virus exposure: a cross-sectional study
Source: BMC Pediatr. 2024 May 17;24:342. doi: 10.1186/s12887-024-04806-y (PMC11100158; doi:10.1186/s12887-024-04806-y)
Supplement: Supplementary file 3 — Supplementary Material 3 [file 12887_2024_4806_MOESM3_ESM.docx]

|  | **1** | **2** | | **3** | | **4** | | **5** | | **6** | | **7** | | **8** | | **9** | | **10** | | **11** | | **12** | | **13** |
| --- | --- | --- | --- | --- | --- | --- | --- | --- | --- | --- | --- | --- | --- | --- | --- | --- | --- | --- | --- | --- | --- | --- | --- | --- |
| 1. BSID-III Cognitive | 1 |  | |  | |  | |  | |  | |  | |  | |  | |  | |  | |  | |  |
| 2. BSID-III Language | 0.75^**^ | 1 | |  | |  | |  | |  | |  | |  | |  | |  | |  | |  | |  |
| 3. BSID-III Motor | 0.70^**^ | 0.82^**^ | | 1 | |  | |  | |  | |  | |  | |  | |  | |  | |  | |  |
| 4. ASQ-3 Communication | 0.64^**^ | 0.76^**^ | | 0.72^**^ | | 1 | |  | |  | |  | |  | |  | |  | |  | |  | |  |
| 5. ASQ-3 Gross Motor | 0.32^*^ | 0.32^*^ | | 0.50^**^ | | 0.44^**^ | | 1 | |  | |  | |  | |  | |  | |  | |  | |  |
| 6. ASQ-3 Fine Motor | 0.57^**^ | 0.64^**^ | | 0.73^**^ | | 0.82^**^ | | 0.53^**^ | | 1 | |  | |  | |  | |  | |  | |  | |  |
| 7. ASQ-3 Problem Solution | 0.65^**^ | 0.71^**^ | | 0.67^**^ | | 0.67^**^ | | 0.30^*^ | | 0.69^**^ | | 1 | |  | |  | |  | |  | |  | |  |
| 8. ASQ-3 Socio Individual | 0.67^**^ | 0.73^**^ | | 0.78^**^ | | 0.84^**^ | | 0.47^**^ | | 0.78^**^ | | 0.67^**^ | | 1 | |  | |  | |  | |  | |  |
| 9. ASQ:SE-2 | -0.58^**^ | -0.47^**^ | | -0.46^**^ | | -0.60^**^ | | -0.19 | | -0.57^**^ | | -0.38^**^ | | -0.61^**^ | | 1 | |  | |  | |  | |  |
| 10. CAPES Intensity | -0.45^**^ | -0.40^**^ | | -0.37^**^ | | -0.49^**^ | | -0.03 | | -0.48^**^ | | -0.36^**^ | | -0.62^**^ | | 0.66^**^ | | 1 | |  | |  | |  |
| 11. CAPES Emotional | -0.49^**^ | -0.47^**^ | | -0.43^**^ | | -0.61^**^ | | -0.12 | | -0.59^**^ | | -0.44^**^ | | -0.59^**^ | | 0.60^**^ | | 0.82^**^ | | 1 | |  | |  |
| 12. CAPES Behavior | -0.42^**^ | -0.36^**^ | | -0.34^*^ | | -0.44^**^ | | -0.002 | | -0.43^**^ | | -0.33^*^ | | -0.59^**^ | | 0.64^**^ | | 0.99^**^ | | 0.74^**^ | | 1 | |  |
| 13. Prenatal Stress | -0.22 | -0.44^**^ | | -0.29^*^ | | -0.27^±^ | | -0.09 | | -0.13 | | -0.29^*^ | | -0.35^*^ | | 0.30^*^ | | 0.47^**^ | | 0.32^*^ | | 0.48^**^ | | 1 |
| Note: **p<0.001; *p<0.05; ^±^p<0.10 | | |  | |  | |  | |  | |  | |  | |  | |  | |  |  |  | |  | |

**Supplementary Table 3. Correlations among the BSID-III, ASQ-3, ASQ:SE-2, CAPES, and prenatal stress.**
